# Adherence to antiretroviral therapy among HIV and AIDS patients at the Kwa-Thema clinic in Gauteng Province, South Africa

**DOI:** 10.4102/phcfm.v8i2.924

**Published:** 2016-06-24

**Authors:** Melaku A. Eyassu, Tebogo M. Mothiba, Nonceba P. Mbambo-Kekana

**Affiliations:** 1 Public Health Unit, University of Limpopo, South Africa; 2 Department of Nursing Science, University of Limpopo, South Africa; 3 Faculty of Health Sciences, University of Limpopo, South Africa

## Abstract

**Background:**

Introduction of antiretroviral therapy (ART) has shown reduction in HIV-related mortality and morbidity in people living with HIV and AIDS. Since high levels of adherence of more than 95.0% is required to achieve effective suppression of viral load, researchers found it important to establish whether people are pursuing what is expected of them.

**Aim and setting:**

The study was aimed at determining adherence to ART among HIV and AIDS patients at the Kwa-Thema clinic in Gauteng Province

**Methods:**

Quantitative cross-sectional descriptive design was used. Ethical clearance was sort from MEDUNSA Research Ethics Committee. Validity and reliability were maintained throughout the study. A non-probability systematic sampling was used. Data were collected using administered structured questionnaire, and a total of 290 respondents were involved. Data were analysed using SPSS software version 22.

**Results:**

The findings indicated that the adherence to ART was 77.0%. Factors that were significantly associated with adherence were gender (*χ*^2^ = 3.78, df = 1, *p* < 0.05), level of education (*χ*^2^ = 3.52, df = 3, *p* = 0.032), co-treatment of HIV and other infections (*χ*^2^ = 5.46, df = 4, *p* = 0.019), ability to follow ART (*χ*^2^ = 12.82, df = 1, *p* = 0.000 < 0.05), and types of antiretroviral drugs.

**Recommendation:**

The study recommends intensification of health education campaign against stigma and gender discrimination. Providing feedback to patients regarding benefits of ART is important.

**Conclusion:**

The study concluded that adherence to ART at the Kwa-Thema clinic was sub-optimal (less than 95%) at 77%, but comparable with the adherence levels in other developing countries.

## Introduction

The human immunodeficiency virus (HIV) and acquired immune deficiency syndrome (AIDS) have become one of the major health problems in many countries in the world. According to UNAIDS 2011, the disease is widely spread in low- and middle-income developing countries, such as South Africa, Botswana and other sub-Saharan African countries. However, the introduction of antiretroviral therapy (ART) brought dramatic changes to the lives of people.^1^ People living with HIV have started to live longer and AIDS-related deaths have also been declining because of the availability of the ART programme. Since 1995, around 2.5 million deaths have been averted in low- and middle-income countries because of increased access to ART.^1^

ART requires a high level of adherence to minimise treatment failure and viral resistance.^2^ There is a very strong relationship between adherence and virologic failure, and research has revealed that an adherence level of more than 95.0% may lead to 22.0% virologic failure, an adherence level between 80.0% and 95% may result in a 61.0% treatment failure and less than or equal to 80.0% adherence may have a treatment failure of 80.0%.^1^ The shift to the use of highly-active antiretroviral therapy (HAART) for treating HIV and AIDS has led to increasingly complex drug regimens.^2,3,4^ These drug regimens present significant challenges to both patients and healthcare providers with respect to adherence.^3^ Without adequate adherence, antiretroviral agents would not be capable of suppressing HIV replication because of insufficient concentrations of drugs in the blood and may lead to difficulties suppressing plasma viral load.^4,5^ In addition to being associated with poor short-term viral response, poor adherence to ART accelerates development of drug-resistant HIV. Therefore, identifying and mitigating the factors that reduce adherence to combination antiretroviral agents are important for prolonged viral load suppression.^4,6^

The number of infected people with HIV has been estimated at around 5.63 million of a population of 46 million.^1,7^ The South African Department of Health indicates that the most common cause of death of HIV and AIDS patients in South Africa is related to non-adherence to ART.^7^ The rate of HIV infection is continually growing in many parts of South Africa, particularly in the Gauteng Province. According to the Department of Health, the KwaZulu-Natal Province has one of the highest spread of HIV infection in South Africa.^5^ In 2008, more than 55.0% of all South Africans infected with HIV resided in the KwaZulu-Natal and Gauteng provinces. The HIV and AIDS prevalence in 2008 in the Gauteng Province was 15.2%. This is an indication that the problem is widely spread.^5,6^ It requires responsibility and hard work to control the spread of HIV in the province. Adherence to ART is one of the approaches that enable healthcare workers to reduce new infections, for example, during pregnancy. This background motivated the researcher to determine adherence to ART at the Kwa-Thema clinic in the Gauteng Province, South Africa.

## Purpose of the study

The study was aimed at determining adherence to ART among HIV and AIDS patients at the Kwa-Thema clinic in Gauteng Province, South Africa.

## Objectives

Describe socioeconomic and demographic factors that are associated with adherence to ART among HIV and AIDS patients at the Kwa-Thema clinic in Gauteng Province, South Africa:

- Identify main factors that hinder adherence to ART among HIV and AIDS patients at the Kwa-Thema clinic.
- Determine the prevalence rate of adherence to ART among HIV and AIDS patients at the Kwa-Thema clinic.

## Research design and methods

A quantitative research approach was adopted using a cross-sectional design. The researchers aimed at establishing factors that influence the adherence to ART among HIV and AIDS patients. A cross-sectional descriptive design was used in this study. This design was chosen so that the factors that determine adherence to ART could be described and understood.^8^

### Study population and sampling method

The study population was all adult HIV and AIDS patients aged above 18 years who were on ART and attending the Kwa-Thema clinic. A systematic sampling method was used for the selection of the participants until the required sample size was obtained. In the clinic, ± 100 patients were attended to per day, and ± 50 patients were started on ART every working day, except on Friday, which is reserved for administration. Respondents were selected randomly (every third patient from the registered book for the day) while waiting to be seen by the clinic doctors. On each day, respondents who met the inclusion criteria were as much as 100 patients who paid the clinic a follow-up visit. After a pill count by the nurse and with limited resources, the investigator invited only the first 15 patients on ART to participate in the study on each day until the required number of 290 was met.

The sample was determined using the following formula of Kothari:^9^

$$n=\frac{Z_{\alpha /2}^{2}\times p\times q}{e^{2}}$$ [Eqn 1]

The total number of respondents in accordance with the above formula was 290. This comprised both males and females.

### Data collection

The researchers collected data for this study using administered structured questionnaire with both open- and closed-ended questions. Researchers were available to clarify questions which were not clear for the respondents during data collection.^10^ Data were collected between 2013 December and 2014 February at the context of this study.

### Data capturing and analysis

Data were analysed using the Statistical Package for Social Science (SPSS) version 22 programme, and the statistical analyses were present in graphic format. These calculations included bivariate (chi-square) and multivariate (logistic regression) analyses that determine correlates or predictors of adherence to ART.

### Ethical considerations

Ethical clearance was obtained from the MEDUNSA Research Ethics Committee (MREC) of University of Limpopo (Ethics Clearance No.: MREC/HS/116/2014:PG and Ekhuruleni Ethics Clearance No.:20/05/2014-1). Written permission to start the study was also received from the Ekurhuleni District Health Ethics Committee. All the ethical principles of informed consent, autonomy, non-maleficence and beneficence, as well as confidentiality were duly observed. Validity and reliability were maintained throughout the study.^8,10^

## Results and discussions

The findings of this study were based on data analysis using the SPSS version 22 programme which includes the following results: Socio-demographic information related to ART adherence, proportion of the respondent adherent to antiretroviral (ARV) treatment, knowledge of the respondents about ART and its influence on ART adherence, treatment regimen, co-management of HIV and other sociocultural factors that influence adherence to ART.

### Gender of respondents

**FIGURE 1:**
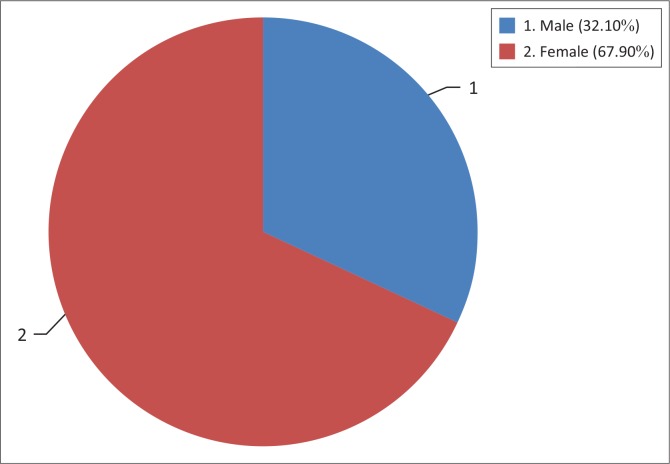
Percentage distribution of gender respondents at the Kwa-Thema clinic.

It was observed that majority of the respondents in this study were females who account for 67.9% as compared with their male counterparts at 32.1%. A further analysis of the respondents’ gender in terms of adherence to ART showed that among female respondents, 80.2% adhered to ART while among the male respondents, 69.9% adhered to treatment (see Figure 1).

### Age of respondents

**FIGURE 2:**
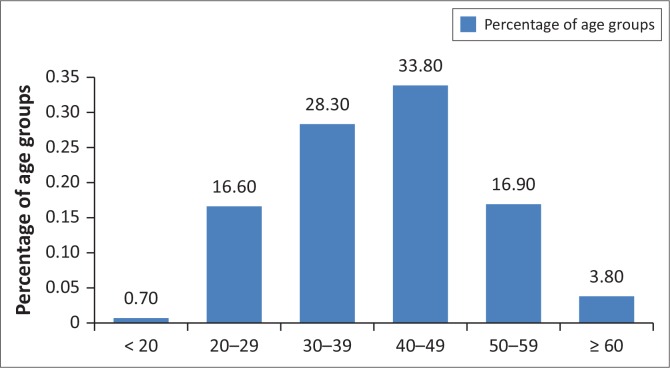
Percentage of respondent age groups at the Kwa-Thema clinic.

The respondents’ age ranged from 18 to older than 60, while the biggest age group was between 40 and 49 years. Most of the respondents were in the reproductive age group of between 20 and 49 years. Respondents in the age group between 50 and 59 years and the group of respondents who were older than 60 years were 16.9% and 3.9%, respectively (see Figure 2).

### Marital status of the respondents

Among the respondents, the majority were single (72.4%), 16.6% were married and others (widowed, cohabitation) made up 11% (see Table 1).

**TABLE 1:** Percentage distribution of marital status of the respondents at the Kwa-Thema clinic.

Marital status	Frequency	Percentage
Single	210	72.4
Married	48	16.6
Other	32	11.0

**Total**	**290**	**100.0**

### Employment status of respondents

**FIGURE 3:**
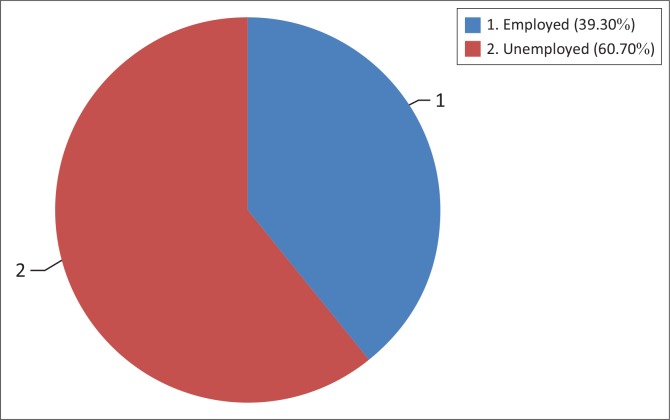
Employed status of respondents at the Kwa-Thema clinic.

The percentage of unemployed respondents was 60.7%, which was very high compared with those employed (39.1%) (see Figure 3).

### Level of education attained by respondents

The percentage distribution of educational status of the respondents is illustrated as follows: The majority of respondents completed secondary school (66.9%), followed by those who had primary school education (20.3%), tertiary education (7.9%) and no formal education (4.8%) (see Table 2).

**TABLE 2:** Education level of the respondents at the Kwa-Thema clinic.

Education level	Frequency	Percentage
No education	14	4.8
Primary	59	20.3
Secondary	194	66.9
Tertiary	23	7.9

**Total**	**290**	**100.0**

### Adherence to antiretroviral therapy based on educational status

A more thorough analysis of adherence status indicated that as education level increases, adherence levels also increase. The adherence level of those at tertiary level was 7.9%, followed by secondary education (66.9%), primary education (20.3%) and the least was noted as the ones without any formal education (4.3%) (see Table 3).

**TABLE 3:** Educational level of the respondents at the Kwa-Thema clinic.

Education level	Frequency	Percentage
No education	14	4.8
Primary	59	20.3
Secondary	194	66.9
Tertiary	23	7.9

**Total**	**290**	**100.0**

### Respondents’ attitude towards antiretroviral treatment, family, friends and community support

**FIGURE 4:**
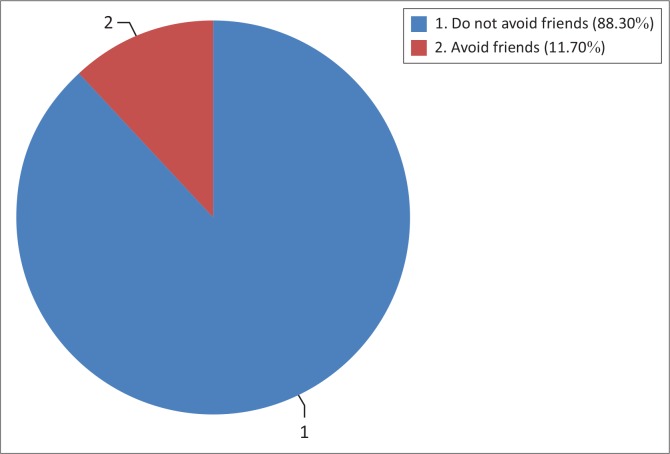
Distribution of respondents according to whether they did or did not receive support from family, friends and community.

All patients (100%) had a positive attitude towards ART and they all approved ART for the management of HIV and/or AIDS. A majority of respondents (88.3%) said that they did not avoid friends or relatives and that they were actually supported by friends during ARV treatment. The rest of the respondents (11.7%) either suffered from stigma, or were not supported by relatives or friends (see Figure 4).

### Knowledge about benefits of antiretroviral therapy

The researcher noted that the majority of respondents (81.4%) knew that ART reduced the viral load and prevented progression to AIDS. The findings revealed that 2.8% of respondents said ART cured HIV and AIDS illness while 8.3% said ART reduced pain. Only 7.6% of respondents said they did not know of any benefits of ART (see Table 4).

**TABLE 4:** Distribution of respondents according to knowledge on benefits of antiretroviral therapy at the Kwa-Thema clinic.

Knowledge of benefits of antiretroviral therapy	Frequency	Percentage
Curing	8	2.8
Reducing pain	24	8.3
Reducing progression	236	81.4
Do not know	22	7.6

**Total**	**290**	**100.0**

### The antiretroviral therapy regimen the respondents were taking

The majority of respondents (67.6%) were taking a TDV-containing regimen followed by a D4t combination (15.9%), and AZT (15.5%). Other types of regimens constituted 1% Regarding the ARV drugs combination the majority of the respondents were getting EFV (77.9%), followed by NVP (18.6%), and other regimens constituted 3.45% (see Table 5).

**TABLE 5:** Distribution of respondents by the types of antiretroviral drugs they were taking.

Antiretroviral drugs	Frequency	Percentage
D4t	46	15.9
TDV	196	67.6
AZT	45	15.5
Others	3	1.0

**Total**	**290**	**100.0**

### Doses of treatment missed and duration of respondents’ treatment

The majority of the respondents (80.7%) did not miss any doses of ARV medication whereas 7.6% missed two doses, and 7.2% missed more than four doses (see Table 6).

**TABLE 6:** Distribution of respondents by doses missed of antiretroviral drugs.

Doses missed	Frequency	Percentage
None	234	80.7
Two	22	7.6
Three	13	4.5
Four and above	21	7.2

**Total**	**290**	**100.0**

### Reason(s) for missing dose(s) of antiretroviral therapy regimen

The study found that 8.3% of respondents who did not adhere to treatment said that they complained about the burden of too many pills, 2.76% felt better and therefore they missed treatment doses, 2.41% said that they missed their treatment doses due to travel or migration to other places, 2.07% said they missed ART doses because they were very ill. The rest of the respondents said they missed their ART due to the side effects of the drugs (1.72%), economic related problems (1.38%), and social stigma (0.67%) (see Table 7).

**TABLE 7:** Distribution of respondents according to reason(s) for missing treatment.

Reasons of missing doses	Frequency	Percentage
Pills burden	24	8.30
Side effect of ARV drugs	5	1.72
Travel/migration	7	2.41
Feels better	8	2.76
Too ill	6	2.07
Economic situations	4	1.38
Stigma	2	0.67
Did not miss	234	80.69

**Total**	**290**	**100.00**

### Source of antiretroviral therapy drugs

The majority of respondents (96.6%) said that government health facilities were the source of ARV drugs whereas 0.7% said that they would get ARV drugs from a chemist when they ran out of ARV drugs, 1.4% said that they would get ARV drugs from friends, and another 1.4% said that they could get ARV drugs from mission hospitals These findings indicated that the majority of respondents (96%) were aware of where they should get their ARV medication (see Table 8).

**TABLE 8:** Distribution of respondents by source of antiretroviral therapy.

Source of antiretroviral drugs	Frequency	Percentage
Chemist	2	0.7
Friends	4	1.4
Public sector	280	96.6
Others	4	1.4

**Total**	**290**	**100.0**

Therefore, ART re-supply was not a problem to patients. No significant association existed between the knowledge about where to get ART supplies and adherence to ART (*χ*^2^ = 3.43, df = 3, *p* = 0.35, > 0.05).

### Co-treatment of HIV and other infections

Most respondents (63.45%) were not being treated for HIV and any other infection at the same time. Nearly a third (29.30%) of the respondents were being treated for other chronic disease, 3.45% of respondents received treatment for co-infection HIV and tuberculosis, 2.07% were treated for a fungal infection, and 1.73 % were being treated for other bacterial infections (see Table 9).

**TABLE 9:** Distribution of respondents according to whether they were undergoing co-infection treatment of HIV and other infections or not.

Other types of infection on Rx	Frequency	Percentage
Tuberculosis treatment	10	3.45
Fungal infection	6	2.07
Antibiotics other than for tuberculosis	5	1.73
Other diseases (specify)	85	29.30
No other treatment	184	63.45

**Total**	**290**	**100.00**

### Distance from the facility

The majority of respondents (90%) had to travel less than 10 km to collect their medication from a clinic. Only 1.4% was collecting their ARV medication by travelling more than 20 km (see Table 10).

**TABLE 10:** Distribution of respondents according to their distance from the clinic.

Distance	Frequency	Percentage
< 5 km	145	50.0
5–10 km	115	39.7
10–20 km	26	9.0
> 20 km	4	1.4

**Total**	**290**	**100.0**

### The influence on adherence to antiretroviral therapy of healthcare facilities and healthcare providers

The majority of respondents (96.6%) said that they were able to follow their ART, 97.63% said that they knew the importance of following the course of treatment strictly and only 2.4% said that they did not know the importance of strictly following the course of ART. Most of the respondents (95.9%) admitted that they were counselled, especially before they had started with ART. Most of the respondents (97.6%) agreed that it was important for HIV-positive patients to be counselled, while they were continuing with ART because it helped to improve ART adherence. Most of respondents (98.6%) said that privacy was maintained during consultations, while only 1.4% said privacy was not maintained.

### Results about the proportion of adherence to antiretroviral therapy

In this study, the ART users’ adherence status was assessed by using self-report assessments for a period of 1 month and pharmacy pill count records by the health providers. The findings showed that self-report assessments indicated that 82.8% adhered to ART while 17.2% did not. With respect to physician assessment, the findings indicate an adherence to ART of 76.9% in comparison with non-adherence of 23.1%.

## Discussion

The discussion of findings is based on data analysis using the SPSS version 22 programme which includes the following results: Socio-demographic information related to ART adherence, proportion of the respondent adherent to ARV treatment, knowledge of the respondents about ART and its influence on ART adherence, treatment regimen, co-management of HIV and other social-cultural factors that influence adherence to ART.

### Gender of the respondents

The findings based on statistical analysis using the Chi-square test of independence showed that there was a significant relationship between the gender of respondents and adherence to ART (*χ*^2^ = 3.78, df = 1, *p* < 0.05). This study has found that females are more likely to attend voluntary counselling and testing (VCT) services and to actively seek healthcare than males. This finding was similar to the finding of Abah et al.^11^ which indicate that in South Africa, the gender of the respondents influenced adherence to ART. Another finding by Nyambura^12^ in Kenya and the Botswana study by Weiser et al.^2^ contain contradicting results that indicate no association between gender and adherence to ART.

### Age of respondents

This finding indicated that the majority of patients at the Kwa-Thema clinic who participated in the study were in the age group between 40 and 49 years (33.8%) and they were more aware of their status and were undergoing ART. However, the results indicated that the age of respondents did not influence ART adherence (*χ*^2^ = 3.5, with df = 5 and *p* = 0.61). Adherence to treatment according to findings of this study was similar in younger and older respondents.

### Marital status of the respondents

The results suggest that unmarried people were the largest category who knew their HIV status and who were taking ARV treatment. However, the chi-square statistic revealed no significant association between marital status of respondents and adherence to ARV treatment (*χ*^2^ = 0.45 with df = 2, *p* = 0.8, > [0.05]).

### Employed status of respondents at the Kwa-Thema clinic

However, these results indicate that statically there was no significant association between occupation of respondents and ART adherence (*χ*^2^ = 1.09 with df = 1, *p* = 0.296). This could be the result easy accessibility to ART and the free provision of the same by the public health sector.

### Adherence to antiretroviral therapy based on educational status

A significant association was noted in this study results between the level of education and adherence to ARV treatment (*χ*^2^ = 3.52; df = 3, *p* = 0.032). These findings are supported by studies on HIV-positive patients in South Africa, Ethiopia, Nigeria and the United States among whom the ones who lack education do not adhere to ART (Abah et al.)^11^ Nyambura.^12^ However, the findings from Botswana by Weiser et al.^2^ indicate that lower levels of education are associated with higher adherence.^13,14,15^

### Respondents’ attitude towards antiretroviral treatment, family, friends and community support

The researchers noted that there was no statistically significant association between adherence to ART and support from family, friends and the community (χ^2^ = 0.25; df = 1, *p* = 0.62). The study in Botswana by Weiser et al.^2^ agrees with the findings of this study, since it reports no significant association between stigma and adherence to ART.

### Knowledge about benefits of antiretroviral therapy

Respondents demonstrated that they did have substantial knowledge about ART. However, no significant association existed between knowledge about ART and adherence (*χ*^2^ = 1.52; df = 3, *p* = 0.68 (*p* > 0.05). This might be because of the fact that despite patients being aware of the benefits of ART and the importance of adherence, there were other factors like support versus lack of support and socioeconomic conditions that led to not adhering to treatment. Most respondents (64.1%) said they had no more frequent sickness; 35.9% were optimistic towards ART and admitted that their CD4+ count (indicated on patients’ cards) improved after taking ARV drugs for at least 1 month. This finding indicated that respondents’ perception of ART was very positive and most of them were knowledgeable about ARV drugs. This finding is supported by other studies.^16,17^ The study in Botswana has found that there is no significant association between side effects of ARV medication and adherence to ART.^2^

### The antiretroviral therapy regimen the respondents were taking

It was found that with regard to the effect of individual ARV drugs that were analysed by means of binary logistic regression, the model selected D4t at *p* = 0.012 and NVP (*p* = 0.021 [< 0.05]) in which the side effects of both drugs were more compared with others.

### Doses of treatment missed and duration of respondents’ treatment

The researcher noted that there was no significant association between the duration and adherence to ART (*χ*^2^ = 4.28; df = 4, *p* = 0.370).

### Reason(s) for missing dose(s) of antiretroviral therapy regimen

The findings confirmed that respondents had various reasons that caused them to miss treatment doses. A significant association existed between reasons for missing treatment doses and adherence to ART (*χ*^2^ = 297 with df = 7, *p* < 0.0001). This finding is supported by studies on AIDS patients in the United States, Canada, Belgium, Brazil and Botswana that show forgetfulness, fear of side effects, feeling better, feeling too sick and pill burden as reasons for ART non-adherence.^16^

### Source of antiretroviral therapy drugs

Therefore, the findings revealed that ART re-supply was not a problem to patients. No significant association existed between the knowledge about where to get ARV supplies and adherence to ART (*χ*^2^ = 3.43, df = 3, *p* = 0.35, > 0.05).

### Co-treatment of HIV and other infections

The findings confirmed that there is a significant association between treatment for both HIV and other infections and adherence to ART (*χ*^2^ = 5.46, df = 4, *p* = 0.019). More than a quarter (29%) of the respondents who had to co-manage HIV and other diseases did not adhere to ART, and they mentioned pill burden as the reason.

### Distance from the facility

The findings revealed that no association was observed between distance from the facility and ART adherence in this study (*χ*^2^ = 1.87; df = 3, *p* = 0.6; since *p* > 0.05).

### The influence on adherence to ART of healthcare facilities and healthcare providers

This finding showed that health care providers adequately informed their patients with regard to their ART medication. A significant association existed between the ability to follow ARV treatment and adherence (*χ*^2^ = 12.82, df = 1, *p* = 0.000, < 0.05).

These findings are supported by a similar study done on HIV patients and healthcare providers where exit interviews, observation, focus group discussion and key informant interviews demonstrate that patients who are adequately informed about ART adhere to it.^11,12^

### Results about the proportion of adherence to antiretroviral therapy

There was no significant difference between self-reporting and health providers’ assessments of ART adherence (Pearson Chi-Square = 180.7, with df = 0.1 and *p* < 0.0001). However, the ART adherence rate of 77% in this study was relatively high compared with findings in developed countries on HIV-positive patients, among whom ART adherence was 55%.^16^

### Recommendations

Based on the findings of the study, the researchers make the following recommendations to enhance the adherence level.

Intensify health education campaigns that address stigma and gender discrimination. Promote family and community support for people living with HIV and AIDS. Provide feedback to patients through healthcare providers about the advantages of ART to their bodies. All of these will encourage them not to skip their medication. Training sessions are required to gain knowledge on disseminating information appropriate to a patient’s level of understanding. It will empower patients with correct knowledge about good adherence practice. Knowledge, talking and showing CD4 count and viral load is therefore very important as both part of training and regular feedback during the time of visits to build trust in the medication. Continuous operational research on adherence since adherence is dynamic, and research is urgently needed to determine patient-important factors for adherence. Although the study indicated relatively high levels of adherence, the development of some guidelines for implementing adherence management strategies are necessary, and they may include the issue of continuous adherence counselling, bringing treatment closer to people, family care model approach to HAART, practical reminders, adherence case management and medication organiser.

## Conclusion

Adherence rates at the Kwa-Thema clinic appeared to be comparable with those in many developed countries despite the fact that patients at the Kwa-Thema clinic faced many structural and economic barriers to treatment. In this study, factors such as gender, education, cultural perceptions and social barriers influenced the level of adherence to the treatment of HIV patients.
